# Impact of the Universal Hepatitis B Immunization Program in Mongolia: Achievements and Challenges

**DOI:** 10.2188/jea.17.69

**Published:** 2007-06-02

**Authors:** Dambadarjaa Davaalkham, Toshiyuki Ojima, Ritei Uehara, Makoto Watanabe, Izumi Oki, Steven Wiersma, Pagvajav Nymadawa, Yosikazu Nakamura

**Affiliations:** 1 Department of Public Health, Jichi Medical University School of Medicine.; 2 Department of Community Health and Preventive Medicine, Hamamatsu University School of Medicine.; 3 Department of Immunization, Vaccines and Biologicals, World Health Organization.; 4 National Center for Communicable Diseases, Mongolia.

**Keywords:** Hepatitis B Virus, Immunization, Program, effectiveness, Epidemiology, Mongolia

## Abstract

**BACKGROUND:**

The impact of the universal infant hepatitis B (HB) immunization program initiated in 1991 in Mongolia is still unclear.

**METHODS:**

A nationwide school-based cross-sectional serosurvey was conducted in 2004, with stratified, multistage, random cluster sampling from all public elementary schools (n=593) in Mongolia. All children were tested for serological markers of hepatitis B virus (HBV).

**RESULTS:**

Serology results were available for 1,145 children (592 boys and 553 girls) aged 7-12 years (survey response rate: 93%). Immunization card was available for 702 (61.3%) children. The coverage of complete HB vaccination was 60.1% and it was increased by birth cohort from 44% to 76%. Significantly higher proportion of children in Metropolitan cities (75.2%) was completely vaccinated with HB compared to those in Province centers (55.7%) and rural areas (59.1%). HBV infection occurred in 5.9%, 13.2%, and 20.8% of complete vaccinees living in Metropolitan, Province centers, and rural areas, respectively; of whom 1.2%, 2.9%, and 8.6% were HB surface antigen (HBsAg) carriers, respectively. Only 17.0% of the children had protective anti-HBs which decreased from 31.1% to 16.3% among 7 to 12-year-olds indicating its decay with time.

**CONCLUSIONS:**

Prevalence of HBV infection and carriage among young generation meaningfully declined compared with those of previous studies in Mongolia. The coverage of birth dose and complete HB vaccination was significantly low in Province centers and rural areas which should be taken into consideration.

Hepatitis B virus (HBV) is a common viral pathogen that has infected an estimated 2 billion people worldwide, including 400 million who have chronic infection. About a quarter of these carriers will develop serious liver disease, including chronic hepatitis, liver cirrhosis and primary hepatocellular carcinoma (HCC), which kill about 500,000-700,000 persons each year.^1^^,^^2^

One of the distinctive features of HBV infection is that the risk of chronicity varies greatly with the age when the infection is acquired. Ninety percent of infected neonates become chronic HBV carriers, as compared with 30% of children infected after the neonatal period but before six years of age. Only 3 to 5% of adults remain chronically infected; the remainders have acute infections resulting in viral clearance.^3^ In high-endemic areas like Mongolia, the most common route of transmission is perinatal, from carrier mother to neonate, or the infection is acquired during early childhood. Moreover, children perinatally infected from their mothers may themselves be a source of horizontal transmission to their younger siblings and playmates.^4^ Thus, the prevention of infection in infants and children through the use of universal infant hepatitis B (HB) vaccination is the most effective strategy to decrease chronic infection and to control the spread of HBV infection worldwide.^5^ Infant HB vaccination has also been proved to reduce the long-term disease burden of HBV, such as HCC.^6^

HBV infection and its sequelae are crucial public health problems in Mongolia. Serological studies conducted earlier among the general population revealed that chronic carriage of HBV and infection is highly endemic in the country.^7^^-^^9^ Consequently, liver cirrhosis and primary HCC are very common among the population (annual incidence rate: 52.1, mortality rate: 46.7 per 100,000 population). For more than one decade, HCC has ranked first in both men and women as a cause of death and morbidity from malignant neoplasm in Mongolia. In addition, it has been estimated that only 8% of the HCC patients were diagnosed at 1st stage of the disease, and more than 75% of cancer cases survived for less than one year after the diagnosis.^10^

To prevent the chronic carrier state and the serious sequelae associated with it, universal infant HB vaccination was introduced into the Expanded Program on Immunization (EPI) of Mongolia in July 1991. However, limited data on HBV infection among the children born after the introduction of this program is available, and no comprehensive assessment has been conducted thus far in Mongolia. Therefore, we carried out a nationwide cross-sectional serosurvey using a representative random sample of elementary school children in order to assess the impacts of universal HB mass vaccination program in decreasing HBV infection and carriage rate in the country and in different residential areas of Mongolia.

## METHODS

### Study Design and Sampling Procedure

We conducted a nationwide school-based serosurvey including all (n=593) public elementary schools in Mongolia. The sampling method has been described in detail elsewhere.^11^^,^^12^ Briefly, a stratified, multistage, probability, random cluster sampling was used to select the study subjects. Sample size was estimated at 994, with an expectation of HBsAg seroprevalence of 3%, ± 1.5% level of accuracy and within 95% confidence limits. The total number of students attending the 2nd grade of all public elementary schools during the academic year of 2003-2004 was used for sample size calculation.

Administratively, Mongolia comprises of the capital city-Ulaanbaatar and 21 provinces (*Aimag* in Mongolian), which are in turn divided into Province centers and surrounding rural counties (known as *Soums*, lower rural administrative units). The territory of Mongolia is divided into 4 main geographical regions. To represent the whole country’s geographic and economic characteristics, we divided Mongolia into 10 strata: Province centers and rural *Soums* in four main geographical regions and capital Ulaanbaatar and non-Ulaanbaatar in Metropolitan areas. Schools were then selected from a list of all schools in the country through multistage, random, cluster sampling with the probability proportional to size of the 2nd grade elementary school population to ensure the self weighting of the sample. As a result, a total of 25 schools were selected in Metropolitan areas, Province centers, and in rural *Soums*. We assumed that 40 children would be selected per cluster.

The Ethical Review Committees of the World Health Organization, Ministry of Health of Mongolian, and Jichi Medical University, Japan approved the study protocol. Parents or guardians of the children received detailed information and written consent was obtained before the study.

### Specimen and Data Collection

The field work was carried out from October through November 2004. Blood samples were obtained for serological testing, separated, and stored at -80°C in the capital, Ulaanbaatar city and Japan, until time of examination.

Information on children’s demographics was recorded from school rosters and parent’s questionnaire. Immunization cards and registries at the local health centers or family hospitals were used to record the vaccination history of each child, because the parent-held immunization card is uncommon in Mongolia. Children who had no immunization card or registry record were considered as “unknown” for vaccination status. Information on HB immunization obtained by interview of parents was not used for the evaluation of the vaccination status due to the questionable validity of the parent’s recollection of children’s HB vaccination after such a long period of time.

### Hepatitis B Vaccination in Mongolia

During 1994-1997, when the majority of the study subjects were born, a plasma-derived vaccine (High Immunogenic Hepatitis B Vaccine, Democratic People’s Republic of Korea) was used in Mongolia. This vaccine was given with a two-dose schedule consisting of first dose within 24-48 hours after birth and second dose at 2 months of age in parallel with tuberculosis vaccine or Bacillus Calmette-Guerin (BCG), oral polio vaccine (OPV), and diphtheria-tetanus-pertussis (DTP) vaccines. In addition, due to remaining stocks of previous years, some children of the above birth cohorts as well as those who were born between 1992 and 1994 were vaccinated using plasma-derived vaccine (3.0 µg, “Hepaccine B”, Cheil, Republic of Korea) with a three-dose schedule at age 0, 2, and 8 months.

### Assessment of Vaccination Coverage

Vaccination coverage for complete HB vaccination was assessed by measuring HB-birth dose, HB2 dose and HB3 dose, and only valid doses were considered. Validity for each HB dose was defined according to the study protocol as the following. Valid HB-birth dose was considered a dose that is given within 24 to 48 hours of birth (within 2 days in this study); valid HB2 is the second dose delivered at least four weeks after HB-birth dose and before the first birthday; and valid HB3 dose (if administered) was considered as the third dose delivered at least four weeks after HB2 and before the first birthday. Vaccination status for complete HB was defined as complete, incomplete and no vaccination. The administration regimen was determined for each child and vaccination status was assessed following the above guidelines.

### Laboratory Analyses

Each serum specimen was analyzed for hepatitis B surface antigen (HBsAg), antibody to hepatitis B surface antigen (anti-HBs) and antibody to hepatitis B core antigen (anti-HBc) by chemiluminescence immunoassay (Abbott^©^ Japan Co.Ltd.) at the SRL Laboratory, Tokyo, Japan. The subjects who were positive for HBsAg only were further tested for IgM anti-HBc by the same method using kits of Abbott^©^ Japan Co.Ltd.

### Interpreting Serological Results

Immunity due to vaccination was considered as the presence of anti-HBs alone with the concentrations of 10 milli-international units per ml (mIU/mL) or higher. Children positive for HBsAg were defined as HBsAg carriers. Any HBV infection included both past and current infections (HBsAg and/or anti-HBc positive).

### Statistical Analysis

Statistical analyses were performed using the Statistical Package for Social Sciences^®^ (Version 15.0, SPSS, Chicago, IL, USA) software. Differences in frequencies were tested by chi-square test or Fisher’s exact test where applicable. The trend in the HB vaccination coverage by year was analyzed using extended Mantel-Haenszel chi-square test for trend. Two-sided P value of less than 0.05 considered to be the level that indicated statistical significance. In the analyses, sampling weight was not incorporated because of the equal probabilities of sample selection.

Because the current study was a cross-sectional study that enrolled children born after the start of mass infant vaccination program among whom the number of unvaccinated subjects was very small, we used the historical references from other studies for comparison with the current study in order to better understand the impact of mass HB vaccination program in Mongolia. The estimated decrease in the prevalence of HBV infection and carriage was calculated using the formula: [(P_unvac_ − P_vac_) ÷ P_unvac_] × 100, or the difference in the prevalence among unvaccinated (P_unvac_) and vaccinated (P_vac_) cases divided by the prevalence of non-vaccinees and multiplied by 100%.

## RESULTS

A total of 1,271 children were selected at random throughout the country, of whom 1,182 subjects agreed to participate in the study (survey response rate: 93.0%). Of these blood samples were not available for 37 students. Finally, a total of 1,145 apparently healthy children (90.1% of the target population, 592 boys and 553 girls) with a mean age of 8.5±0.7 years (range: 7 to 12 years) were tested for all serological markers of HBV. These children constituted nearly 2.0% of the total Mongolian students in the 2nd grade of elementary schools. Four hundred and thirty three children (37.8%) of the study populations were from Metropolitan areas, 193 (16.9%) were from central towns of the Provinces, and the remaining subjects (45.3%) were from rural *Soums*.

Based on the interpretation of HBV marker results, the following profiles were observed in the study subjects. One hundred and twenty two children (10.7%) had anti-HBs and anti-HBc without HBsAg-positivity, 56 children (4.9%) were positive for both HBsAg and anti-HBc (IgG), 2 children (0.2%) were HBsAg-positive but negative for anti-HBc of IgG and IgM classes, 195 children (17.0%) were anti-HBs-positive only, 769 children (67.2%) were negative for all markers and a child (0.1%) had all markers of HBsAg, anti-HBs and anti-HBc.

The coverage of HB vaccination in the country and comparison by residential areas are shown in Table 1. The immunization card was available for 702 (61.3%) children, which was more available in rural areas than in urban settings. Children who had immunization documentation were slightly younger than those who did not (p=0.021). Among those with immunization cards, 422 (60.1%) children were completely vaccinated with HB vaccine. Nearly 65% of the children had received the birth doses on time, whereas the remaining subjects received the birth dose late (31.9%) or birth doses of HB were not administered (3.4%). The coverage of timely given birth vaccine was significantly lower in Province centers (59.8% vs. 75.2%, p=0.010) and in rural areas (63.1% vs. 75.2%, p=0.006) compared with Metropolitan cities. Similarly, the complete HB vaccine coverage was more likely to be lower in Province centers (55.7% vs. 68.0%, p=0.047) and rural *Soums* (59.1% vs. 68.0%, p=0.036) than in Metropolitan areas. However, as combined there were no significant differences in the coverage of valid birth HB vaccine (p=0.230) and complete HB vaccination (p=0.466) between urban and rural areas.

**Table 1.** Characteristics of study population and coverage of hepatitis B (HB) vaccination by residence.

Variables	Total (n=1,145)	Urban (n=626)			

		Metropolitan	Province centers	Subtotal	Rural *Soums*
		(n=433)	(n=193)	(n=626)	(n=519)
Age, mean (SD)^†^	8.5 (0.8)	8.5 (0.8)	8.3 (0.7)	8.5 (0.8)	8.6 (0.8)
Sex, boys^†^	592 (51.7)	225 (52.0)	104 (53.9)	329 (52.6)	263 (50.7)
Immunization card, Yes^†^	702 (61.3)	125 (28.9)	122 (63.2)	247 (39.5)	455 (87.7)^***^
HB vaccination status^‡^					
HB-Birth vaccine					
Valid	454 (64.7)	94 (75.2)	73 (59.8)	167 (67.6)	287 (63.1)
Late	224 (31.9)	26 (20.8)	37 (30.3)	63 (25.5)	161 (35.4)^**^
No vaccine	24 (3.4)	5 (4.0)	12 (9.8)	17 (6.9)	7 (1.5)
HB-Total vaccine					
Complete	422 (60.1)	85 (68.0)	68 (55.7)	153 (61.9)	269 (59.1)
Incomplete	271 (38.6)	37 (29.6)	52 (42.6)	89 (36.0)	182 (40.0)
Unvaccinated	9 (1.3)	3 (2.4)	2 (1.6)	5 (2.0)	4 (0.9)

Variables are shown as number and proportion, n (%).

**p<0.01; ***p<0.001 compared to the corresponding values in Urban areas (subtotal).

† : Distributions of age, sex and immunization card are shown for all children (n=1,145).

‡ : HB vaccination status is shown for those (n=702) who have immunization documentation.

SD: Standard deviation

Table 2 shows the prevalence of HBV infection and carriage by residence and the HB vaccination status. Among the 1,145 elementary school children 15.8% had been infected with HBV, of whom 5.2% were HBsAg carriers. The corresponding values among complete vaccinees were 16.6% and 6.2%, respectively. The prevalence of HBV infection and carriage varied significantly among residential areas: HBV infection occurred in 5.9%, 13.2%, and 20.8% of complete vaccinees living in Metropolitan cities, Province centers, and rural areas, respectively; of whom 1.2%, 2.9%, and 8.6% were HBsAg carriers, respectively. The differences in the frequencies of HBV infection (p=0.002) and HBsAg carriage (p=0.007) between urban and rural areas were statistically significant. Incomplete vaccinees as well as children with unknown status for HB vaccination also showed similar trends for HBV infection and HBsAg carrier status in regard to residential areas.

**Table 2.** Prevalence of hepatitis B virus (HBV) infection and carriage among 1,145 elementary school children by residence and vaccination status against hepatitis B.

HBV infection and vaccination status	Total (n=1,145)	Urban (n=626)			Rural *Soums* (n=519)

		Metropolitan	Province centers	Subtotal	
Total HBV infection					
Complete	70/422 (16.6)	5/85 (5.9)	9/68 (13.2)	14/153 (9.2)	56/269 (20.8)^**^
Incomplete	53/271 (19.6)	3/37 (8.1)	9/52 (17.3)	12/89 (13.5)	41/182 (22.5)
Not vaccinated	1/9 (11.1)	0/3 —	0/2 —	0/5 —	1/4 (25.0)
Unknown^†^	57/443 (12.9)	32/308 (10.4)	13/71 (18.3)	45/379 (11.9)	12/64 (18.8)
Total	181/1145 (15.8)	40/433 (9.2)	31/193 (16.1)	71/626 (11.3)	110/519 (21.2)^***^
HBsAg carriage					
Complete	26/422 (6.2)	1/85 (1.2)	2/68 (2.9)	3/153 (2.0)	23/269 (8.6)^**^
Incomplete	16/271 (5.9)	0/37 —	3/52 (5.8)	3/89 (3.4)	13/182 (7.1)
Not vaccinated	0/9 —	0/3 —	0/2 —	0/5 —	0/4 —
Unknown	17/443 (3.8)	12/308 (3.9)	1/71 (1.4)	13/379 (3.4)	4/64 (6.3)
Total	59/1145 (5.2)	13/433 (3.0)	6/193 (3.1)	19/626 (3.0)	40/519 (7.7)^***^

Variables are shown as number and proportion, n (%).

**p<0.01; ***p<0.001 compared to the corresponding values in Urban areas (Subtotal).

† : Vaccination status was considered as “unknown” for those who had no immunization documentations.

Among the study population anti-HBs was present in 300 children, of whom 195 (17.0%) had antibody induced by HB vaccine (Table 3). The vaccine induced anti-HBs-positivity rates in Metropolitan areas, Province centerss and rural Soums, were 19.6%, 15.5%, and 15.4%, respectively. One child with unvaccinated status was observed to be positive for anti-HBs alone, which probably indicates misclassification of HB immunization due to inaccurate recording. The vaccine-induced immunity did not significantly vary between majority (*Khalkh*) and minority (non-*Khalkh*) ethnic groups as well as between boys and girls (data not shown).

**Table 3.** Prevalence of vaccine-induced anti-HBs antibody among study population by residence and vaccination status against hepatitis B.

HB vaccination status	Total (n=1,145)	Urban (n=626)			Rural *Soums* (n=519)

		Metropolitan	Province centers	Subtotal	
Complete	67/422 (15.9)	16/85 (18.8)	11/68 (16.2)	27/153 (17.6)	40/269 (14.9)
Incomplete	41/271 (15.1)	5/37 (13.5)	5/52 (9.6)	10/89 (11.2)	31/182 (17.0)
Not vaccinated	1/9 (11.1)	0/3 —	0/2 —	0/5 —	1/4 (25.0)
Unknown	86/443 (19.4)	64/308 (20.8)	14/71 (19.7)	78/379 (20.6)	8/64 (12.5)
Total	195/1145 (17.0)	85/433 (19.6)	30/193 (15.5)	115/626 (18.4)	80/519 (15.4)

Variables are shown as number and proportion, n (%).

**Figure 1.**
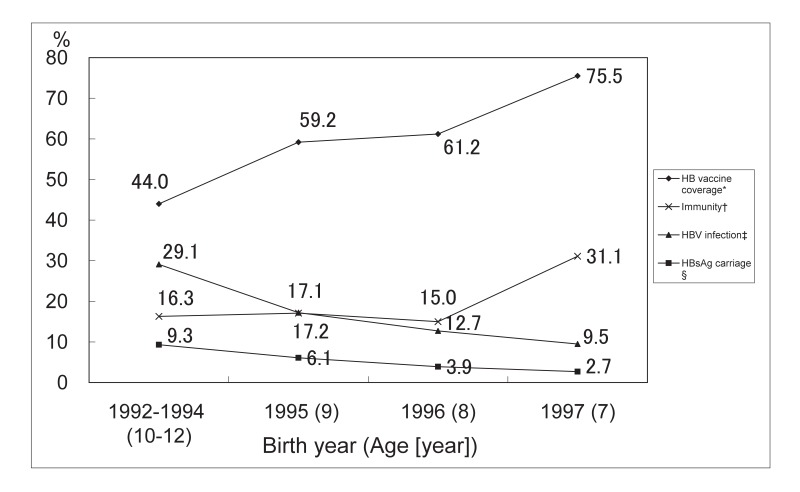
Coverage rate of complete hepatitis B (HB) vaccination, prevalence of hepatitis B virus (HBV) infection, and vaccine-induced immunity among study population by year of birth * : The coverage of complete HB vaccination among the study population † : Immunity induced by HB vaccine (only anti-HBs-positive) ‡ : Past HBV infection and carriage § : Positive for HBsAg

As shown in Figure 1, the coverage of complete HB vaccination in the study subjects increased between 1992 and 1997 from 44.0% to 75.5% (p for trend 0.004). The proportion of children with vaccine induced protective anti-HBs levels (10+ mIU/mL) was decreased by age from 31.1% to 16.6%. In contrast, the frequencies of HBV infection and HBsAg carriage were increased by age, and were higher in older cohorts than in younger cohorts.

## DISCUSSION

In this survey, using a representative random sample of vaccinated cohorts we assessed the impact of the universal infant HB vaccination program in Mongolia. To our knowledge, this is the first nationwide survey to evaluate the mass HB vaccination program since its introduction in 1991 in Mongolia. The age, sex and ethnic distributions of the study subjects were similar to population in the second grade in the country.^13^ In addition, the geographic distribution of the children included in this study were widely distributed throughout the country, and therefore the results should be considered representative for Mongolia.

As with the national trend of increasing coverage of HB vaccine since 1991, the complete vaccination coverage among the study population also increased by birth cohort during the 6 year period (from 1992 through 1997). The higher rate of HBV infection and HBsAg carriage in the older children could be associated with this increasing coverage of HB vaccine over the years. Compared to the reported coverage rates in the country (47.4% and 88.5%, respectively)^10^ the estimates in this study were slightly lower. These differences could be attributed to the methodology of using only valid doses in estimation of the vaccination coverage among study subjects in this study.

Worldwide, many endemic countries of HBV infection have introduced universal immunization of infants, with subsequent success in reducing the incidence of HBsAg carriage.^14^^-^^16^ According to the studies conducted among the population born before the era of mass infant HB vaccination program, nearly 80% of the general population was infected with HBV and up to 15% were carriers of this virus in Mongolia.^7^^-^^10^ Although the study reports among children were very limited, prevalence of HBV infection in unvaccinated children were reported to be 31% and HBsAg carriage 10-13.6%.^17^^,^^18^ In order to better estimate the impact of mass HB vaccination program in the country, we used the above historical references from other studies (HBV infection-31% and HBsAg carriage-13.6%^17^^,^^18^) to compare the results of the current study (total HBV infection-15.8% and HBsAg carriage-5.2%; Table 2) by examining the overall declines in the HBV infection and carriage rates among children. It has been found that the prevalence of HBV infection and carriage have decreased by 49% and 62%, respectively in Mongolia. In terms of residence, the declines in the HBV infection and carriage were higher in urban areas (64% and 78%, respectively) than in rural areas (32% and 43%, respectively).

The significant urban-rural differences in the prevalence of HBV infection and carriage suggest some potential gaps in the vaccination program implementation in the remote areas. It should be noted that there were no significant differences in the risk of infection or in the frequency of HBV-infected mother among urban and rural subjects (7.3% vs. 9.6%, p=0.194).^19^ One of the potential causes may include improper vaccine storage and handling leading to the vaccine damage during the cold winter season of Mongolia. It is well known that the HB vaccine loses its immunological potency upon freezing or freeze drying.^20^

Indeed, in our recent study,^19^ it has been observed that HB vaccines could have damaged during the transport and storage in cold winter season particularly in the remote areas of Mongolia. In the multivariate regression model, those who received all HB vaccines in winter had more than two fold significantly increased risks of being infected with HBV or being HBsAg carriers. In contrary, the frequency of vaccine-induced immunity was significantly lower among children with winter administration of birth HB vaccine than in those with non-winter administration (11.3% vs. 20.6%; p=0.007) in rural areas but not in urban settings (17.4% vs. 13.6%, p=0.468). A cold-chain study by Edstam et al^21^ has also shown an exposure of HB vaccine to freezing temperatures during the transport in rural areas in Mongolia. The freezing of HB vaccine was explained by transporting vaccines with ice packs taken directly from deep freezes at -20°C, but the environmental or seasonal effect has not been considered in this study.

In high-endemic areas of HBV infection, a major route of transmission is perinatal and timing of the birth dose appeared to be the most important factor in achieving protective efficacy and to prevent perinatal HBV transmission.^22^ In this study, the coverage of timely given birth HB vaccine was significantly lower in rural areas and Province centers compared to Metropolitan cities, whereas in contrast the late administration of birth HB was more likely to be common. Consistent to our observations, significantly lower rate of timely given birth dose and complete HB vaccination in rural 2-year-olds compared to Metropolitan subjects were previously reported in Mongolia.^23^

Long-term follow-up studies conducted in other countries have demonstrated that the level of anti-HBs antibody does wane after vaccination, quite rapidly within the first years and more slowly thereafter.^24^^-^^26^ For instance, in Alaska, USA, where HBV infection was also endemic, the vaccine induced anti-HBs-positivity declined from 100% in the first year to 19% in the 5 years and 8% in the 10 years of age.^27^ Similar decreasing trend of detectable anti-HBs with age was found in the present study; the vaccine-induced immunity decreased from 31.1% to 16.3% among 7 to 10-12-year olds.

The potential limitation of the current study could be low immunization card availability in urban areas particularly in Metropolitan cities compared to rural areas, which may lead to the bias and decrease in the power of some statistical analysis. The potential bias may be due to different socioeconomic statuses of those with and without immunization documentation. However, according to the results in Metropolitan cities, there were no significant differences between children with and without immunization documentation in the distributions of socioeconomic variables including father’s (p=0.104) and mother’s occupation (p=0.280), and family size (p=0.99). In addition, it should be noted that immunization cards and registries are not held by parents, but they are kept at the local health centers or family hospitals in Mongolia. Therefore, it is assumed that the low availability of immunization documentation in Metropolitan area might not have caused a bias.

Despite the above limitation, we assessed the impact of mass HB vaccination program in Mongolia using representative random sample of vaccinated generation. The current study was the first nationwide survey that was carried out after the introduction of mass HB vaccination in the country. Therefore, the results of this study would be helpful to better understand the current situation of HBV infection among the vaccinated generation and the vaccination program implementation.

In conclusion, the results of the current survey show low prevalence of HBV infection and carriage among young generation who were born after the start of the mass vaccination program compared to those rates reported previously in Mongolia. Although the vaccination coverage increased over the years in the country, it is essential to increase the coverage of timely given birth dose and complete HB vaccination in Province centers and rural areas of Mongolia.
